# Superconducting Nd_1−*x*_Eu*_x_*NiO_2_ thin films using in situ synthesis

**DOI:** 10.1126/sciadv.adh3327

**Published:** 2023-07-05

**Authors:** Wenzheng Wei, Dung Vu, Zhan Zhang, Frederick J. Walker, Charles H. Ahn

**Affiliations:** ^1^ Department of Applied Physics, Yale University, New Haven, CT 06520, USA.; ^2^ Advanced Photon Source, Argonne National Laboratory, Argonne, IL 60439, USA.; ^3^ Department of Physics, Yale University, New Haven, CT 06520, USA.; ^4^ Department of Mechanical Engineering and Materials Science, Yale University, New Haven, CT 06520, USA.

## Abstract

We report on superconductivity in Nd_1−*x*_Eu*_x_*NiO_2_ using Eu as a 4f dopant of the parent NdNiO_2_ infinite-layer compound. We use an all–in situ molecular beam epitaxy reduction process to achieve the superconducting phase, providing an alternate method to the ex situ CaH_2_ reduction process to induce superconductivity in the infinite-layer nickelates. The Nd_1−*x*_Eu*_x_*NiO_2_ samples exhibit a step-terrace structure on their surfaces, have a *T*_c_ onset of 21 K at *x* = 0.25, and have a large upper critical field that may be related to Eu 4f doping.

## Teaser

Rare earth atom substitution produces a nickelate superconductor.

## INTRODUCTION

Nickelate superconductivity has become the subject of intense research efforts following the synthesis of Sr:NdNiO_2_ (*1*). After the initial discovery, other compositions of infinite-layer nickelates, e.g., Sr:PrNiO_2_, Ca:LaNiO_2_, and Sr:LaNiO_2_, have been shown to be superconducting (*2*–*5*). Two key factors in the superconducting nickelates are a nominal hole-doping level of 20% by alkaline earth substitution of the lanthanide site and a redox reaction to form the infinite-layer structure. Hole doping for these superconducting nickelates is achieved using a 2+ alkaline earth substitution or additional 2+ rare earth rock salt layer (*6*), and redox reaction by ex situ CaH_2_ anneal process (*7*). Here, we describe an approach to dope a rare earth–only nickelate, Nd_1−*x*_Eu*_x_*NiO*_y_* (NENO), to induce superconductivity using an in situ reduction method (*8*).

The approach harnesses the idea of orbital engineering (*9*) involving the 4f electronic configuration of Eu (*10*). Atomic Eu has a [Xe]4f^7^6s^2^ configuration, so the half-filled 4f^7^ orbitals stabilize 2+ europium in rock salt EuO (*11*), while the 3+ valence is also observed in Eu_2_O_3_. As a dopant, Eu^2+^ shows notable similarities with Sr^2+^, from similar ionic size, as shown by similar lattices in EuTiO_3_ and SrTiO_3_ (*12*), to a large difference between the second and third ionization energies (*13*). We expect that the half-filled 4f configuration in Eu^2+^ increases the third ionization energy of Eu and stabilizes the 2+ configuration. These observations suggest Eu as a candidate for doping NdNiO_2_.

We apply an in situ metal reduction method (*8*) to synthesize the mixed–rare earth superconducting infinite-layer nickelate Nd_1−*x*_Eu*_x_*NiO_2_. We show that the Eu substitution produces a nickelate superconductor with a maximal onset critical temperature (*T*_c_) of 21 K at *x* = 0.25 Eu composition and infer a large out-of-plane upper critical field by measurement of field-dependent data taken to 14 T. We also compare Nd_1−*x*_Eu*_x_*NiO_2_ to its alkaline earth–doped counterpart Nd_1−*x*_Sr*_x_*NiO_2_ and find that the charge transfer from Ni to Eu in Nd_1−*x*_Eu*_x_*NiO_2_ is not as complete as from Ni to Sr in Nd_1−*x*_Sr*_x_*NiO_2_. The superconductivity in Nd_1−*x*_Eu*_x_*NiO_2_ and its differences with Nd_1−*x*_Sr*_x_*NiO_2_ should stimulate additional experiments and theory, which may further reveal the role of 4f electrons in superconducting nickelates.

## RESULTS AND DISCUSSION

### In situ synthesis and doping mechanism of NENO

**Fig. 1.**
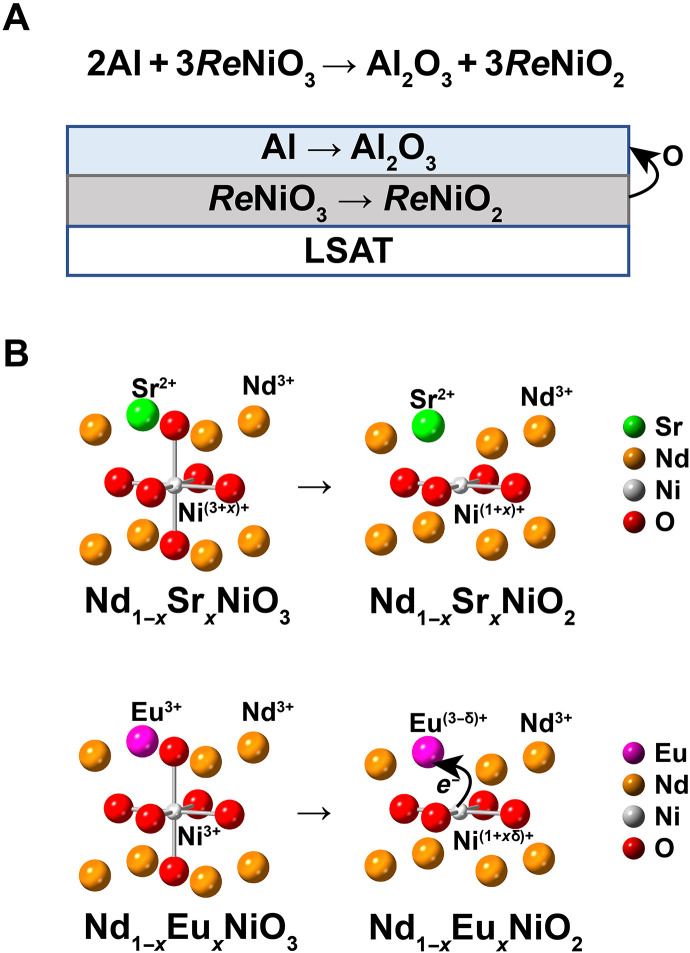
Schematic diagram of oxygen and charge transfer before and after redox reaction. (**A**) Redox reduction happens between the Al capping layer and rare earth perovskite nickelate (*Re*NiO_3_). (**B**) A comparison of Nd_1−*x*_Sr*_x_*NiO*_y_* (NSNO) and NENO before and after reduction shows the charge transfer effect in NENO. Note that Ni is Ni^(3+*x*)+^ in Nd_1−*x*_Sr*_x_*NiO_3_ and is Ni^3+^ in Nd_1−*x*_Eu*_x_*NiO_3_ films, whereas Eu is Eu^3+^ in Nd_1−*x*_Eu*_x_*NiO_3_ and Eu^(3-δ)+^ in Nd_1−*x*_Eu*_x_*NiO_2_.

The synthesis of the parent compound Nd_1−*x*_Eu*_x_*NiO_3_ has been well studied (*14*). Thin films of Nd_1−*x*_Eu*_x_*NiO_3_ are grown to a thickness of 18 to 21 unit cells (uc) on (001)-oriented (LaAlO_3_)_0.3_(Sr_2_AlTaO_6_)_0.7_ (LSAT) substrates using molecular beam epitaxy (MBE). The subsequent reduction for NENO is achieved by in situ reduction using an Al capping method described in (*8*). The reduction method minimizes hydrogen intercalation effects (*15*, *16*). The schematic reaction diagram is shown in Fig. 1A. The selection of LSAT instead of SrTiO_3_ as a substrate is meant to suppress the role of the substrate in the thin-film redox reaction (*17*) and to reduce stacking defects observed in infinite-layer nickelate synthesis (*18*, *19*).

A schematic diagram of charge transfer in the Nd_1−*x*_Sr*_x_*NiO*_y_* (NSNO) and NENO systems is shown in Fig. 1B. In this diagram, the parent compound with Sr dopants means that the Ni needs to have a valence higher than 3+ for the stoichiometric compound (*7*, *20*) and may be the reason why the parent compound can be difficult to synthesize (*7*). This high valence of Ni is avoided in Nd_1−*x*_Eu*_x_*NiO_3_ due to the stability of the Eu^3+^, which allows Ni to remain in the 3+ oxidation state. In this sense, Eu acts as a charge reservoir as its valence changes during growth and reduction. Because the synthesis of EuNiO_3_ is easier than SrNiO_3_ (*21*), exploring the phase diagram in the overdoped (*x* > 0.5) infinite-layer nickelates may be facilitated.

### Surface and structural characterization for NENO

**Fig. 2.**
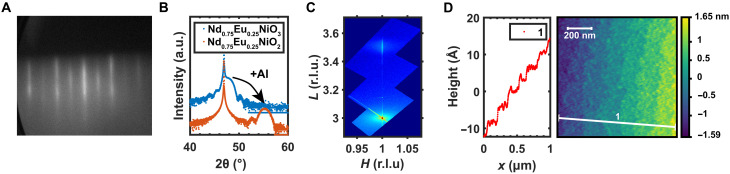
Surface and structural characterization of NENO samples. (**A**) Reflection high-energy electron diffraction pattern of a 20-uc-thick *x* = 0.25 Nd_1−*x*_Eu*_x_*NiO_3_ sample shows a 2 × 2 reconstruction pattern. (**B**) A shift of the (002) diffraction peak is observed after Al reduction indicating complete reduction. a.u., arbitrary units. (**C**) Measurements of the off-specular (103) reciprocal space map (RSM) of an *x* = 0.25 Nd_1−*x*_Eu*_x_*NiO_2_ sample indicate that the film is coherently strained. The axes are in LSAT reciprocal lattice units. *L*, out-of-plane reciprocal lattice parameter; *H*, in-plane reciprocal lattice parameter; r.l.u., reciprocal-lattice units.. (**D**) Atomic force microscopy image and line profile on an Al-capped *x* = 0.25 Nd_1−*x*_Eu*_x_*NiO_2_ thin film. The root mean square roughness is 1.88 Å.

Structural and morphology characterizations of NENO samples are shown in Fig. 2. A 20-uc-thick *x* = 0.25 Nd_1−*x*_Eu*_x_*NiO_3_ thin film shows a clear 2 × 2 reconstruction reflection high-energy electron diffraction pattern (Fig. 2A). The reconstruction pattern is commonly observed in perovskite nickelates and may indicate NiO_6_ octahedra rotations (*22*–*24*). X-ray diffraction (XRD) characterization of an *x* = 0.25 Nd_1−*x*_Eu*_x_*NiO_3_ sample shows an out-of-plane lattice constant of 3.78 Å. After solid-state reduction with Al, this lattice constant decreases to 3.31 Å (Fig. 2B) due to the removal of apical oxygen from the rare earth layer, as is commonly observed in the synthesis of square-planar nickelates (*5*, *6*, *25*, *26*). After reduction, the Nd_1−*x*_Eu*_x_*NiO_2_ film remains strained to the LSAT substrate, as seen from the off-specular reciprocal space map (RSM) shown in Fig. 2C. The surface of an Al-capped Nd_1−*x*_Eu*_x_*NiO_2_ sample shows step terraces with a root mean square roughness of 1.88 Å and an average step height of ~4 Å (Fig. 2D), in line with the substrate lattice parameter. The structural and morphology characterization shows that the Al_2_O_3_-Nd_1−*x*_Eu*_x_*NiO_2_ and Nd_1−*x*_Eu*_x_*NiO_2_-LSAT interfaces remain abrupt after reduction (*8*). See fig. S1 for additional structural characterization and fig. S2 for more surface morphology characterization.

### Electrical characterization for NENO

**Fig. 3.**
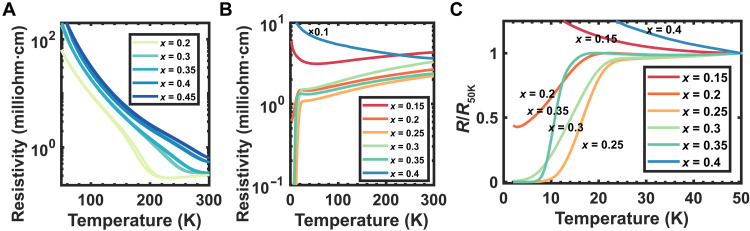
Electrical characterization of NENO samples. (**A**) Resistivity-versus-temperature behavior of Nd_1−*x*_Eu*_x_*NiO_3_ samples shows a metal-to-insulator transition at a temperature that depends on Eu concentration *x*. (**B**) Resistivity-versus-temperature curves (2 to 300 K) of Nd_1−*x*_Eu*_x_*NiO_2_ samples show superconductivity in the doping level 0.2 < *x* < 0.35. The resistivity for the *x* = 0.4 sample is multiplied by a factor of 0.1. Resistivity-versus-temperature curves between 2 and 50 K are plotted in (**C**) and are normalized at 50 K.

Electrical transport results of NENO samples are shown in Fig. 3. For Nd_1−*x*_Eu*_x_*NiO_3_ samples, a metal-to-insulator transition is observed (Fig. 3A). The transition occurs at 230 K in an *x* = 0.2 Nd_1−*x*_Eu*_x_*NiO_3_ sample and at 280 K in an *x* = 0.3 sample, consistent with previous reports (*27*).

The temperature dependence of the resistivity for Nd_1−*x*_Eu*_x_*NiO_2_ samples is shown in Fig. 3 (B and C) for a doping range of 0.15 to 0.4. Films with *x* between 0.2 and 0.35 are metallic and show a resistance drop at 10 to 21 K. Superconducting behavior is observed for *x* = 0.25, *x* = 0.3, and *x* = 0.35 (Fig. 3B). The highest superconducting transition temperature is 21 K for onset and 9.5 K for zero resistance, observed for *x* = 0.25 Nd_1−*x*_Eu*_x_*NiO_2_ samples (Fig. 3C). This transition is broader than that for Nd_1−*x*_Sr*_x_*NiO_2_ films (12.8-K onset to 7.5-K zero resistance) (*28*). See fig. S3 for further low-temperature transport characterization and fig. S4 for current-voltage characteristics from an *x* = 0.25 Nd_1−*x*_Eu*_x_*NiO_2_ sample.

**Fig. 4.**
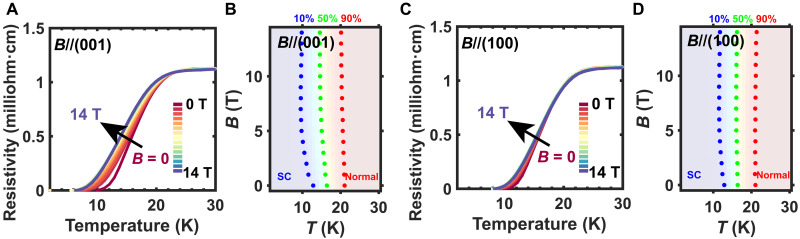
Superconducting transition in an *x* = 0.25 Nd_1−*x*_Eu*_x_*NiO_2_ as a function of magnetic field. (**A**) Low-temperature resistance of *x* = 0.25 Nd_1−*x*_Eu*_x_*NiO_2_ as a function of out-of-plane magnetic field. The direction of magnetic field is along the (001) direction. Note that the onset temperature of superconductivity changes from 21 K in zero field to 20.4 K when *B* = 14 T is applied. The onset temperature is defined as the temperature at which the resistivity drops to 90% of the normal resistivity just above *T*_c_. *B*, magnetic field. (**B**) Out-of-plane *B*-*T* phase diagram of *x* = 0.25 Nd_1−*x*_Eu*_x_*NiO_2_ is produced from transport data shown in (A). Note that the upper critical field where superconductivity disappears is much higher than the highest measured field of 14 T. SC, superconducting. (**C**) Low-temperature resistance of *x* = 0.25 Nd_1−*x*_Eu*_x_*NiO_2_ as a function of in-plane magnetic field. The direction of magnetic field is along the (100) direction. (**D**) In-plane *B*-*T* phase diagram of an *x* = 0.25 Nd_1−*x*_Eu*_x_*NiO_2_ thin film is produced from transport data from (C).

The temperature-dependent magnetoresistance of an *x* = 0.25 Nd_1−*x*_Eu*_x_*NiO_2_ superconducting film (Fig. 4A) indicates that the superconductivity is suppressed by an out-of-plane magnetic field. The change of superconducting transition temperature as a function of applied out-of-plane magnetic field is shown in Fig. 4B. Compared to the broad transition temperature range (12.8 to 21 K for 10 to 90% resistance temperature), the influence of magnetic field on transition temperature (1.6 K difference for the 50% resistance temperature from 0 to 14 T) is relatively small. In the Nd_1−*x*_Eu*_x_*NiO_2_ system, the upper critical fields are several times higher than the highest applied field of 14 T (Fig. 4B). The magnetoresistance shows a smaller change with the in-plane magnetic field (Fig. 4, C and D), and the magnetoresistance becomes negative at high field for *x* = 0.3 and *x* = 0.35 Nd_1−*x*_Eu*_x_*NiO_2_ samples. See fig. S3 for more field-dependent electrical characterization. An anomalous magnetoresistance and violation of the Pauli limit represent an interesting phenomenon observed in other superconducting nickelate systems (*28*), with the violation of the Pauli limit being interpreted in several ways. One interesting possibility is that it is related to a different pairing mechanism in the Nd_1−*x*_Eu*_x_*NiO_2_ system (*28*). It may also suggest an intrinsic magnetism in superconducting Nd_1−*x*_Eu*_x_*NiO_2_ films. This suggestion is supported by the observation of magnetic excitations in other infinite-layer nickelate superconductors (*29*, *30*). Divalent Eu 4f^7^ is in a high spin state (*31*), which may complicate the magnetic ground state of the Ni in superconducting Nd_1−*x*_Eu*_x_*NiO_2_ thin films. In the Nd_1−*x*_Sr*_x_*NiO_2_ system, the superconducting transition broadens when applying a magnetic field (*28*); the broad transition of Nd_1−*x*_Eu*_x_*NiO_2_ films and weak dependence of *T*_c_ on external field may indicate magnetic contributions from Eu. More work is needed to understand the role of Eu in the superconducting Nd_1−*x*_Eu*_x_*NiO_2_ thin films and its magnetoresistance behavior as a superconductor. The superconducting transition is notably robust up to our highest measured field of 14 T. Because of the small change in *T*_c_, additional measurements at higher field are necessary.

### Role of rare earth orbitals on charge transfer

We also discuss the most direct effect of Eu in Nd_1−*x*_Eu*_x_*NiO_2_, which is charge transfer. Two charge transfer mechanisms are likely as follows: One is Ni-3d to rare earth 5d (*Re*-5d) charge transfer, and the other is Ni-3d to Eu-4f transfer. The *Re*-5d mechanism is widely discussed in the *Re*NiO_2_ compounds, also known as a self-doping effect (*5*, *32*, *33*). Because of this effect, *Re*-5d orbitals can accommodate electrons from Ni-3d electrons after topotactic reduction and serve as “charge reservoirs” (*34*). In NdNiO_2_, this effect can hole-dope NiO_2_ planes by 0.08 electrons/uc (*33*, *35*–*37*). However, the doping level associated with this effect is small and shows little influence on the superconducting phase diagram of infinite-layer nickelates (*18*, *38*), and the 3d^8.8^ electron configuration on Ni is still achieved by alkaline doping (*6*).

Similar to Ni-3d to *Re*-5d hybridization (*18*, *38*), the charge transfer between Ni-3d and Eu-4f can be viewed as a self-doping effect. A question arises as to how much is changed by this effect on the NiO_2_ doping level, i.e., δ in Fig. 1B. Three possibilities are considered here. One is the δ = 0 case: Ni-3d to Eu-4f self-doping does not modify the doping level of NiO_2_, analogous to the Ni-3d to *Re*-5d charge transfer shown in NdNiO_2_. This possibility is ruled out by the observation of superconductivity in Nd_1−*x*_Eu*_x_*NiO_2_ samples. Another possibility is the δ = 1 case, in which complete charge transfer occurs between Ni and Eu so that Eu is completely 2+ in the film, acting the same as an alkaline earth dopant. The third case is a hybridized case, in which an Eu f orbital lies at the Fermi level so that a partial charge transfer occurs when 0 < δ < 1.

**Fig. 5.**
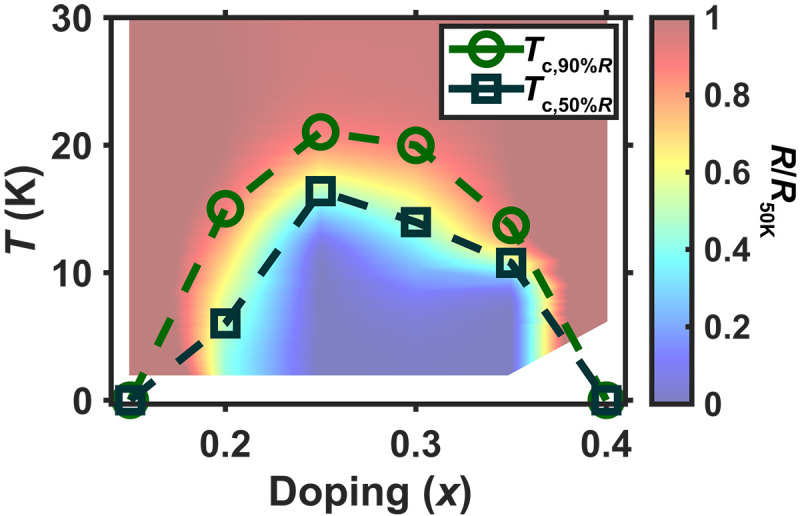
The superconducting phase diagram of Nd_1−*x*_Eu*_x_*NiO_2_. The phase diagram shows that superconductivity is achieved at 0.15 < *x* < 0.4, a higher doping level *x* for Eu than that for Sr substitution [0.1 < *x* < 0.3 in (*39*)], so the charge transfer from Ni to Eu in Nd_1−*x*_Eu*_x_*NiO_2_ is weaker than that in Sr in Nd_1−*x*_Sr*_x_*NiO_2_. A comparison of superconducting temperature between Nd_1−*x*_Eu*_x_*NiO_2_ samples and their Sr analogs is hindered by a possible thickness dependence for nickelate superconducting temperature (*2*). We note, however, that all data in this work are obtained on films with the same thickness (18 to 21 uc). The dashed lines connect the 90% (onset) and 50% residual resistivity (half-transition) temperatures for samples of different doping levels to define a contour for the superconducting dome. The color map shows the relative resistivity from Nd_1−*x*_Eu*_x_*NiO_2_ samples of different doping levels normalized to 50 K. The statistical error for *T*_c_ is on order of 1 K. The relative error in doping level *x* is limited by flux measurement and is on order of 1%.

To visualize these three possibilities, we plot the superconducting phase diagram of Nd_1−*x*_Eu*_x_*NiO_2_ as a function of doping level *x* (Fig. 5) and compare the phase diagram with Nd_1−*x*_Sr*_x_*NiO_2_ (*39*). We find that the superconducting dome shifts toward larger *x* in Nd_1−*x*_Eu*_x_*NiO_2_ (0.15 < *x* < 0.4) compared to Nd_1−*x*_Sr*_x_*NiO_2_ [0.1 < *x* < 0.3 in (*39*)]. The shift in the superconducting dome indicates that the Eu ions contribute fewer holes than Sr ions at the same doping level, so the valence of Eu ions in the Nd_1−*x*_Eu*_x_*NiO_2_ films should be between Eu^2+^ and Eu^3+^. Therefore, 0 < δ < 1 and a partly filled 4f orbital from Eu crossing the Fermi surface is expected in Nd_1−*x*_Eu*_x_*NiO_2_. The partial charge transfer between Ni-3d and Eu-4f orbitals indicates that the bonds between Ni and Eu ions are more covalent than that between Ni and Sr.

**Fig. 6.**
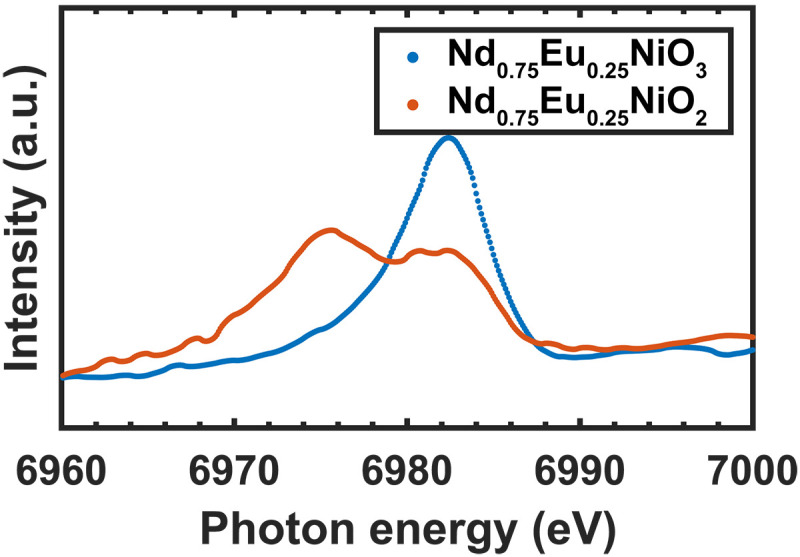
Diffraction XANES from NENO at Eu L3 edge. A single Eu^3+^ peak appears in the spectrum from a Nd_0.75_Eu_0.25_NiO_3_ film, and an additional Eu^2+^ peak shows up ~7 eV below Eu^3+^ peak in a Nd_0.75_Eu_0.25_NiO_2_ sample.

To determine δ, we perform diffraction x-ray near edge absorption spectroscopy (XANES) at the Eu L3 edge to selectively measure the spectrum from the perovskite and square planar phases. This measurement is sensitive to Eu valence due to a large 7-eV chemical shift of the white line between Eu^2+^ and Eu^3+^ (*40*, *41*). The results are shown in Fig. 6, where we compare the spectra for NENO samples. In Nd_1−*x*_Eu*_x_*NiO_3_, a single white line peak is observed with a peak energy consistent with Eu^3+^. When reduced to Nd_1−*x*_Eu*_x_*NiO_2_, an additional peak appears at lower energy characteristic of Eu^2+^. This result reveals mixed Eu^2+^ and Eu^3+^ valences on the A-site cation, i.e., 0 < δ < 1. Fitting the two peaks gives δ = 0.6, so we find that Eu^(3−δ)+^ substitution for *x* = 0.25 Nd_1−*x*_Eu*_x_*NiO_2_ is equivalent to a Sr^2+^ doping level of *x* = 0.15 in Nd_1−*x*_Sr*_x_*NiO_2_. Temperature-dependent resonant inelastic x-ray scattering on the soft Ni L and O K edges will provide insight into doping induced changes in the NiO_2_ plane (*42*).

In summary, we modify existing synthetic routes to superconducting nickelates in two ways: We apply an in situ Al reduction approach to synthesize an infinite-layer nickelate and achieve hole doping with a Ni-3d Eu-4f self-doping mechanism. We rule out an exclusive effect of hydrogen intercalation (*43*) and find a prototype superconducting nickelate system with a *T*_c_ of 21 K. The Nd_1−*x*_Eu*_x_*NiO_2_ system shows an unusually high upper critical field and a shift of the *T*_c_ doping phase diagram, indicating a more complex role of rare earth 4f electrons on superconductivity. We find that doping of the NiO_2_ planes likely arises from a charge reservoir effect shown as a change of the average Eu valence in NENO. Finding NENO provides important alternative synthetic routes and may provide unique insight into unconventional superconductivity.

## MATERIALS AND METHODS

### Sample growth

Thin films of Nd_1−*x*_Eu*_x_*NiO_3_ are grown to a thickness of 18 to 21 uc on as-received commercial 5 mm–by–5 mm LSAT (001) substrates (from CrysTec) using MBE. The substrates are cleaned at 605°C by using an activated radio-frequency plasma oxygen source at a chamber pressure of 8 × 10^−6^ torr for 15 min before thin-film synthesis. The thin films are grown under the same conditions as for the plasma cleaning process. After growth, the films are cooled in activated oxygen to 150°C to eliminate oxygen vacancy formation. Some thin films are transferred in air for ex situ characterization, and others are kept in the chamber for in situ reduction using metallic Al as described in (*8*).

### Sample characterization

XRD and RSMs of the samples are taken using a rotating anode high-resolution x-ray diffractometer (Rigaku SmartLab). The x-ray energy is fixed at the Cu-Kα energy of 8.04 keV. Electrical transport characterization is conducted in a Quantum Design Dynacool PPMS using the van der Pauw measurement geometry for resistivity. Superconducting onset temperature is defined as the temperature at which the resistivity drops to 90% of the normal state resistivity just above *T*_c_. Diffraction XANES measurements are performed at the beamline 33-ID-D of the Advanced Photon Source, Argonne National Laboratory. The intensity modulation of the (001) diffraction peak across the Eu L3 edge is fitted with an iterative Kramers-Kronig algorithm (*44*–*46*) to get the XANES spectra.
