# Supplementary material for: Superconducting Nd1−xEuxNiO2 thin films using in situ synthesis
Source: Sci Adv. 2023 Jul 5;9(27):eadh3327. doi: 10.1126/sciadv.adh3327 (PMC10321736; doi:10.1126/sciadv.adh3327)
Supplement: Supplementary file 1 — Figs. S1 to S4 [file sciadv.adh3327_sm.pdf]

Science Advances
AAAS

Supplementary Materials for

Superconducting $Nd_{1-x}Eu_xNiO_2$ thin films using in situ synthesis

Wenzheng Wei *et al.*

Corresponding author: Charles H. Ahn, charles.ahn@yale.edu; Wenzheng Wei, wenzheng.wei@yale.edu

Sci. Adv. **9**, eadh3327 (2023)
DOI: 10.1126/sciadv.adh3327

This PDF file includes:

Figs. S1 to S4

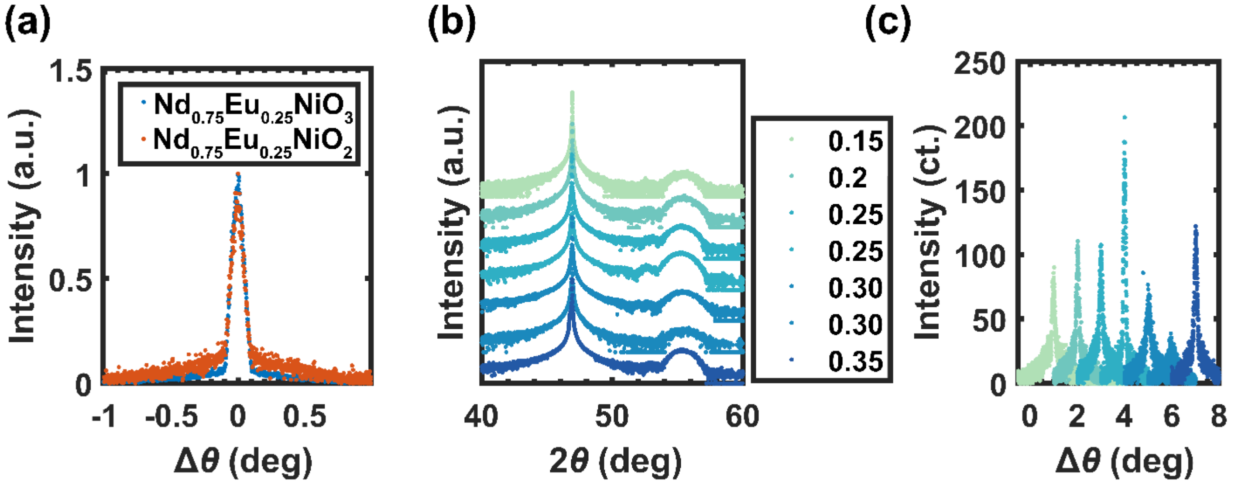

Fig. S1 supplemental X-ray diffraction characterization on NENO samples. (**a**) A comparison of rocking curves shows the same peak width between a $Nd_{1-x}Eu_xNiO_3$ and $Nd_{1-x}Eu_xNiO_2$ thin film (x = 0.25). The curves are normalized to their maxima. The FWHM for both peaks is 0.135°. (**b**) X-ray diffraction θ-2θ scan on $Nd_{1-x}Eu_xNiO_2$ samples (x from 0.15 to 0.35) show 002 diffraction maxima. (**c**) Rocking curves of 002 diffraction on samples shown in (**b**). The intensity is in units of counts from the detector and show the 002-diffraction peak intensity. Each curve is shifted by an additional degree in the same sequence shown in (**b**).

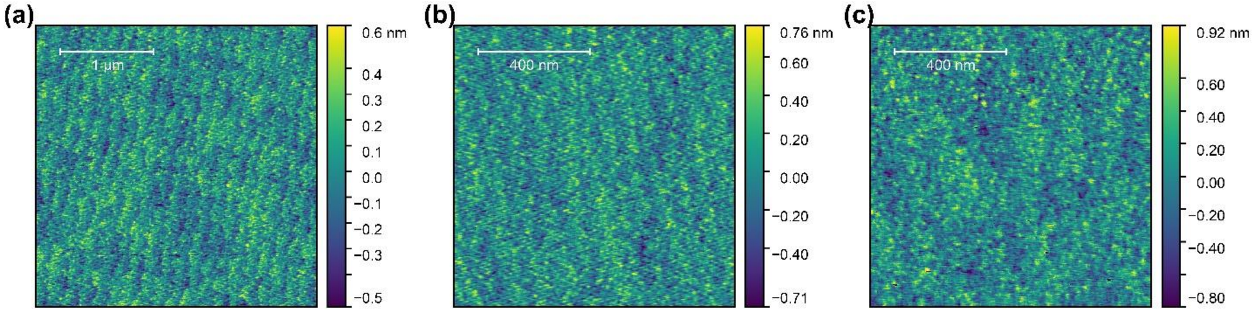

Fig. S2 surface morphology by AFM. (**a**) 3 × 3 um AFM image shows an RMS of 1.85Å on the same x = 0.25 $Nd_{1-x}Eu_xNiO_2$ sample shown in **Fig.2D**. (**b**) 1 × 1 um AFM image shows an RMS of 2.32 Å on an x = 0.3 $Nd_{1-x}Eu_xNiO_2$ sample. (**c**) 1 × 1 um AFM image shows an RMS of 2.81 Å on an x = 0.35 $Nd_{1-x}Eu_xNiO_2$ sample.

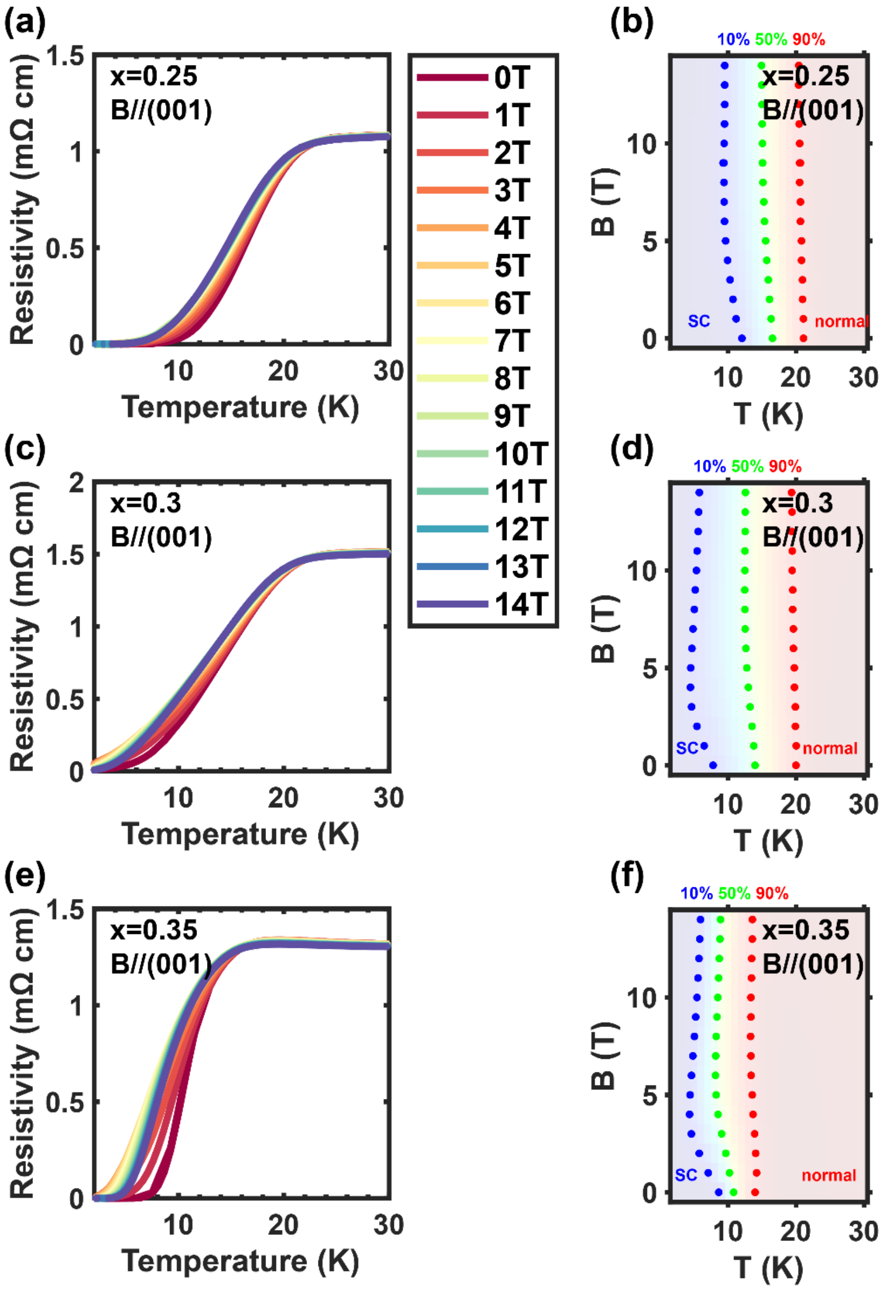

Fig. S3 Additional electrical characterization on superconducting NENO samples shows magnetoresistance behaviors. (**a,c,e**) are resistivity-temperature curves measured on $Nd_{1-x}Eu_xNiO_2$ samples of different doping levels x (x = 0.25,0.3 and 0.35 respectively). (**b,d,f**) are the corresponding phase diagrams.

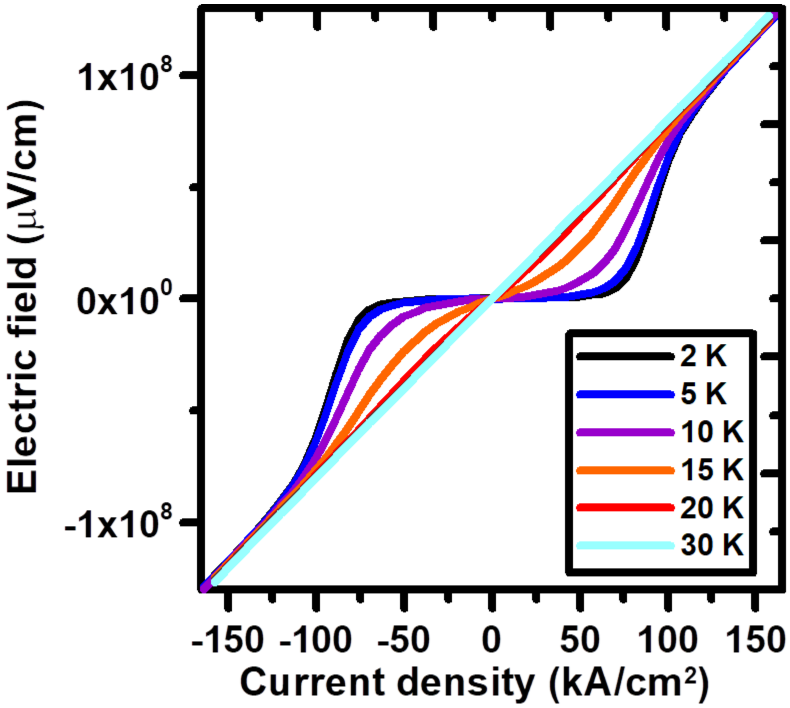

Fig. S4 Critical current density characterization on an x = 0.25 $Nd_{1-x}Eu_xNiO_2$ sample at various temperatures.
